# Intermittent versus continuous androgen deprivation for locally advanced, recurrent or metastatic prostate cancer: a systematic review and meta-analysis

**DOI:** 10.1186/1471-2490-14-9

**Published:** 2014-01-25

**Authors:** Tobias Engel Ayer Botrel, Otávio Clark, Rodolfo Borges dos Reis, Antônio Carlos Lima Pompeo, Ubirajara Ferreira, Marcus Vinicius Sadi, Francisco Flávio Horta Bretas

**Affiliations:** 1 Evidencias Scientific Credibility, Campinas, São Paulo, Brazil; 2 Comitê Brasileiro de Estudos em Uro-Oncologia (CoBEU), São Paulo, Brazil

**Keywords:** Androgen deprivation, Prostate cancer, Systematic review

## Abstract

**Background:**

Prostate cancer is the most common cancer in older men in the United States (USA) and Western Europe. Androgen deprivation (AD) constitutes, in most cases, the first-line of treatment for these cases. The negative impact of CAD in quality of life, secondary to the adverse events of sustained hormone deprivation, plus the costs of this therapy, motivated the intermittent treatment approach. The objective of this study is to to perform a systematic review and meta-analysis of all randomized controlled trials that compared the efficacy and adverse events profile of intermittent versus continuous androgen deprivation for locally advanced, recurrent or metastatic hormone-sensitive prostate cancer.

**Methods:**

Several databases were searched, including MEDLINE, EMBASE, LILACS, and CENTRAL. The endpoints were overall survival (OS), cancer-specific survival (CSS), time to progression (TTP) and adverse events. We performed a meta-analysis (MA) of the published data. The results were expressed as Hazard Ratio (HR) or Risk Ratio (RR), with their corresponding 95% Confidence Intervals (CI 95%).

**Results:**

The final analysis included 13 trials comprising 6,419 patients with hormone-sensitive prostate cancer. TTP was similar in patients who received intermittent androgen deprivation (IAD) or continuous androgen deprivation (CAD) (fixed effect: HR = 1.04; CI 95% = 0.96 to 1.14; p = 0.3). OS and CSS were also similar in patients treated with IAD or CAD (OS: fixed effect: HR = 1.02; CI 95% = 0.95 to 1.09; p = 0.56 and CSS: fixed effect: HR = 1.06; CI 95% = 0.96 to 1.18; p = 0.26).

**Conclusion:**

Overall survival was similar between IAD and CAD in patients with locally advanced, recurrent or metastatic hormone-sensitive prostate cancer. Data on CSS are weak and the benefits of IAD on this outcome remain uncertain. Impact in QoL was similar for both groups, however, sexual activity scores were higher and the incidence of hot flushes was lower in patients treated with IAD.

## Background

Prostate cancer is the most common cancer in older men in the United Kingdom (UK), United States (USA) and Western Europe [1]. Despite its high incidence, the disease is often responsive to treatment even when metastatic and may be cured when localized. In patients with locally advanced tumors, recurrent or metastatic, the goals of therapy are to prolong survival, slow the progression of disease and preserve the quality of life [2].

Androgen deprivation (AD) constitutes, in most cases, the first-line of treatment for these cases. However, the deleterious effects of continuous androgen deprivation (CAD) are widely known and are related to some degree of deterioration in the quality of life. The most frequent symptoms resulting from AD include sexual dysfunction, fatigue, anemia, reduced muscle and bone mass, depression, abnormal lipid metabolism, cognitive dysfunction and development or worsening of metabolic syndrome [3,4]. The negative impact of CAD in quality of life, secondary to the adverse events of sustained hormone deprivation, plus the costs of this therapy, motivated the intermittent treatment approach.

In recent years, non-randomized studies (most of them with heterogeneous criteria for selection and clinical assessment) were performed to confirm the effectiveness of intermittent androgen deprivation (IAD) in patients with prostate cancer. Two systematic reviews [5,6] previously published analyzed the results of these non-randomized studies and the authors suggested that the best candidates for intermittent androgen deprivation are patients with biochemical progression after prostatectomy or radiation, with no evidence of metastases and with mildly aggressive tumors. On the other hand, patients with large tumor volumes, positive lymph nodes and bone metastases, PSA >100 ng/ml or short PSA doubling time, would be best treated with continuous deprivation.

Irrespective of official guideline recommendations, IAD is a treatment option used worldwide by both urologists and oncologists even outside of clinical trials [5].

The 2008 UK National Institute for Health and Clinical Excellence (NICE) recommends that IAD may be offered to men with metastatic prostate cancer providing they are informed that there is no long-term evidence of its effectiveness [7].

The results of some randomized controlled trials (RCT) were statistically inconclusive [8] or controversial. Crook et al. [9,10] analyzed IAD versus CAD for prostate-specific antigen (PSA) elevation after radiotherapy, assessing overall survival (OS) in a non-inferiority randomized trial with 1,386. The results demonstrated that IAD was non-inferior to CAD in regard to OS (8.8 years versus 9.1 years respectively - hazard ratio for death, 1.02; CI 95% 0.86 to 1.21, p for non-inferiority = 0.009), besides providing potential benefits in aspects such as physical function, fatigue, urinary problems, hot flashes, libido, and erectile function. Furthermore, the authors pointed that time to hormone resistance was statistically significantly improved on the IAD arm (HR 0.80, 95% CI 0.67-0.98; p = 0.024).

Regarding time-to-progression or castration-resistant disease, however, Crook et al. [9,10] showed better results in favor of CAD, while the results of Salonen et al. [11,12] were favorable to IAD. Such contradictory results make it difficult to emit a strong scientific-based recommendation for or against IAD.

The objective of this study is to analyze all published randomized controlled trials (RCTs) that compared the efficacy and adverse events profile of IAD versus CAD for locally advanced, recurrent or metastatic hormone-sensitive prostate cancer.

## Methods

### Study selection criteria

#### Types of studies

We included RCTs with parallel design that compared the use of intermittent versus continuous androgen deprivation.

#### Types of participants

The selected studies included patients with locally advanced, recurrent or metastatic hormone-sensitive prostate cancer.

### Search strategy for identification of studies

A wide search on the main computerized databases was conducted, including EMBASE, LILACS, MEDLINE, SCI, CENTRAL, The National Cancer Institute Clinical Trials service and The Clinical Trials Register of Trials Central. In addition, the abstracts published in the proceedings of the American Society of Clinical Oncology (ASCO), American Society of Radiation Oncology (ASTRO), the European Society of Medical Oncology (ESMO), Society of Urologic Oncology (SUO), European Society for Radiotherapy and Oncology (ESTRO) and American Urological Association (AUA) were also searched.

For MEDLINE, we used the search strategy methodology for randomized controlled trials [13] recommended by the Cochrane Collaboration [14]. For EMBASE, adaptations of this same strategy were used [13], and for LILACS, we used the search strategy methodology reported by Castro et al. [15]. An additional search on the SCI database was also performed to retrieve articles that were cited on the included studies. The specific terms relevant to this review were added to the overall search strategy methodology for each database.

The overall search strategy was: #1: prostate cancer, #2: intermittent, #3: androgen deprivation and #4: Randomized Controlled Trial. Searches in electronic databases combined the terms: #1 AND #2 AND #3 AND #4.

The search was not limited by date, language or specific outcome.

### Critical evaluation of the selected studies

All the references retrieved by the search strategies had their title and abstract evaluated by two of the researchers. Every reference with the least indication of fulfilling the inclusion criteria was listed as pre-selected. The complete article of all pre-selected references was retrieved. The articles were analyzed by two different researchers and included or excluded according to the previously reported criteria. The excluded trials and the reason of their exclusion are listed in this article. Data was extracted from all the included trials.

Details regarding the main methodology characteristics empirically linked to bias [16] were extracted with the methodological validity of each selected trial assessed by two reviewers (T.E.A.B and O.C). Particular attention was given to some items such as: the generation and concealment of the sequence of randomization; blinding; application of intention-to-treat analysis; sample size pre-definition; loss of follow-up description; adverse events reports; if the trial was performed in multiple center or a single center; and the sponsorship.

### Data extraction

Two independent reviewers extracted the data. The name of the first author and year of publication were used to identify the study. All data were extracted directly from the text or calculated from the available information when necessary. The data of all trials were based on the intention-to-treat principle, so they compared all patients allocated in one treatment with all those allocated in the other.

The primary endpoint was overall survival (OS). The OS was calculated from the date of randomization to the date of death, with data censored at the last known date that the patient was alive.

Other clinical outcomes were also evaluated:

• Cancer-specific survival (CSS): the cancer-specific survival was calculated from the date of randomization to the date of death from prostate cancer or a complication of cancer treatment;

• Time to progression (TTP) or castration-resistant disease: defined as increases in PSA level or evidence of new clinical disease while the patient was receiving androgen-deprivation therapy and testosterone was at castration level;

• Differences in the quality of life (QoL): hot flashes, desire for sexual activity, urinary symptoms, depression and gynecomastia;

• Died because of cardiovascular events.

### Analysis and presentation of results

Data analysis was performed using the Review Manager 5.1.2 statistical package (Cochrane Collaboration Software) [17].

Dichotomous clinical outcomes are reported as Risk Ratio (RR) and survival data as Hazard Ratio (HR) [18]. The corresponding 95% confidence interval (CI 95%) was calculated, considering *P* values less than 5% (p < 0.05). Statistic Heterogeneity was calculated through I2 method (25% was considered low-level heterogeneity, 25-50% moderate-level heterogeneity and >50% high-level heterogeneity) [19,20].

To estimate the absolute gain in OS, cancer-specific survival and time to progression, the meta-analytic survival curves were calculated as suggested by Parmar et al. [18]. A pooled estimate of the HR was computed by a fixed-effect model according to the inverse-variance method [21]. Thus, for effectiveness or adverse events, an HR or RR >1 favors the standard arm (continuous treatment) whereas an HR or RR <1 favors the intermittent treatment.

If statistic heterogeneity was found in the meta-analysis, an additional analysis was performed, using the random-effects model described by DerSimonian and Laird [22] that provides a more conservative analysis.

To assess the possibility of publication bias, the funnel plot test described by Egger et al. was performed [23]. When the pooled results were significant, the number of patients needed to treat (NNT or NNH) to cause or to prevent one event was calculated by pooling absolute risk differences in the trials included in this meta-analysis [24-26]. For all analyses, a forest plot was generated to display the results.

## Results

**Figure 1:**
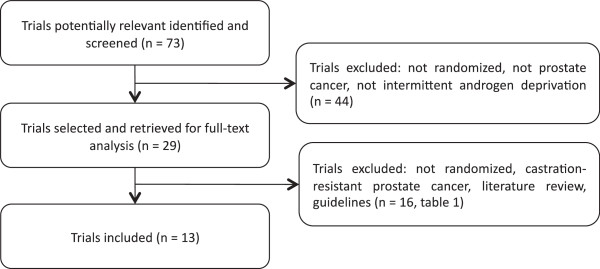
Trial selection flow.

The diagram represents the flow of identification and inclusion of trials, as recommended by the PRISMA (Preferred Reporting Items for Systematic reviews and Meta-Analyses) statement [27] (Figure 1).

Overall, 73 references were identified and screened.

Twenty-nine studies were selected and retrieved for full-text analysis. Of these studies, 16 were excluded for various reasons described on Table 1.

**Table 1:** Characteristics of excluded studies

**Study**	**Reason for exclusion**
Klotz [28]	Not a randomized trial
Goldenberg [29]	Not a randomized trial
Higano [30]	Not a randomized trial
Oliver [31]	Not a randomized trial
Bierkens [32]	Not a randomized trial
Grossfeld [33]	Not a randomized trial
Calais da Silva Jr F [34]	Analysis of pooled data from 2 trials
De Conti P [35]	Systematic Review
Loblaw [36]	Practice guideline
Heidenreich [37]	Practice guideline
Heidenreich [38]	Not a randomized trial
Heidenreich [39]	Practice guideline
Buchan [40]	Not a randomized trial
Keizman [41]	Not a randomized trial
Gruca [42]	Literature review
Organ [43]	Castration-resistant prostate cancer

### Characteristics of included studies

Thirteen randomized trials comprising 6,419 patients with hormone-sensitive prostate cancer were included in this analysis. More than 2,800 patients (44%) had metastatic disease and 1,595 patients (25%) had PSA progression after prostatectomy (n = 209) or radiotherapy (n = 1,386). The number of patients in these trials varied from 43 to 1,535, but only 5 involved >500 patients. The average age of patients was 70 years.

PSA was measured every 2-3 months in most studies [1,9-12,44-54]. Only in one study [8,55] PSA levels were measured monthly.

Full details of trial design are not available for all studies: several reports are available only in abstract form [48,49,56].

The criteria for stopping and re-start of therapy in groups treated with IAD were similar, however not uniform (Table 2). Average follow-up time in these studies was of >2 years. The induction period varied, but most patients used IAD for a period of approximately 3-6 months (Table 3).

**Table 2:** Characteristics of randomized included studies

**Study**	**Patients with PCa**	**N (ITT)**	**PSA levels (ng/ml)**		**Primary endpoint**	**Median follow-up (years)**
			**Cease treatment**	**Restarted treatment**		
Crook 2012 [9,10]*(CIC-CTG PR.7 Trial)*	Recurrent (after radiotherapy)	1386	<4 and not > 1 above the previous recorded value as monitored	>10	OS	6.9
Calais da Silva 2009/2011 [44,45]*(SEUG Trial)*	Locally advanced, metastatic	626	<4 ≤80% initial level	≥10 (symptomatic) or ≥20 (asymptomatic) ≥20% above nadir value	TTP	4.75
Hussain 2013 [8,55]*(SWOG 9346 Trial)*	Metastatic	1535	≤4	≥20 or ≥10 or symptoms	OS	9.2
Salonen 2012/2013 [11,12]*(FinnProstate Trial VII)*	Locally advanced, metastatic or recurrent (after radiotherapy or prostatectomy)	554	<10 >50% (PSA < 20)	PSA or CP	TTP	5.4
Tunn 2012 [1,46,47]*(EC507 Trial)*	Recurrent (after prostatectomy)	201	≤0.5	≥3 or when was CP	Time to androgen-independent	2.4
De Leval 2002 [51]	Locally advanced, metastatic or recurrent (after prostatectomy)	68	≤4	≥10	Time to androgen- independent	2.7
Langenhuijsen 2008/2011 [52,53] (*TULP Trial*)	Locally advanced or metastatic	193	<4	≥10 (M0) ≥20 (M1)	TTP	2.58
Miller 2007 [56]	Locally advanced or metastatic	335	<4	-	TTP	NR
			≤90% initial level			
Mottet 2012 [50]*(TAP 22 Trial)*	Metastatic and PSA ≥ 20 ng/ml	173	< 4	≥10 or CP	OS	3.7
Verhagen 2008/2013 [48,49]	Asymptomatic metastatic	258	Good or moderate response	PSA or CP	Quality of life	NR
Hering 2000 [54]	Metastatic	43	0.4	≥10 (initial ≤20)	TTP and adverse events	4
				± 50% initial (initial > 20)		
Irani 2008 [57]	Locally advanced or metastatic	129	6 months	6 months	Quality of life and TTP	5
Silva 2013 [58]*(SEUG 9901 Trial)*	Locally advanced or metastatic	918	< 4	≥20 or CP	OS	5.5

*Abbreviations*: *OS* overall survival, *TTP* time to progression, *PCa* prostate cancer, *PSA* prostate-specific antigen, *CP* clinical progression, *NR* not reported, *ITT* intent-to-treat, *CP* clinical progression.

**Table 3:** Treatment regimens in the included studies

Crook 2012 *(CIC-CTG PR.7 Trial)*[9,10]	***Intermittent:*** induction only (8 mo): LHRHa injections plus a non-steroidal antiandrogen, with the latter continued for a minimum of 4 weeks.
	***Continuous:*** consisted of a LHRHa plus a non-steroidal antiandrogen, with the latter continued for a minimum of 4 weeks, or orchiectomy.
Calais da Silva 2009/2011 *(SEUG 9401 Trial)*[44,45]	***Intermittent:*** induction only (3 mo): CPA 200 mg for 2 weeks followed by monthly depot injections of a LHRHa plus 200 mg of CPA daily.
	***Continuous:*** received an LHRHa plus 200 mg of CPA daily.
Hussain 2013 *(SWOG 9346 Trial)*[8,55]	***Intermittent:*** induction only (7 mo): received LHRHa (goserelin) plus bicalutamide.
	***Continuous:*** LHRHa plus bicalutamide
Salonen 2012/2013 *(FinnProstate Study VII)*[11,12]	***Intermittent:*** induction only (6 mo): goserelin acetate (3.6 mg) SC every 28 days. The CPA was given in 100 mg twice daily during the first 12.5 days to minimize flare reaction.
	***Continuous:*** continued with goserelin acetate or bilateral orchiectomy.
Tunn 2012 *(EC507 Trial)*[1,46,47]	***Intermittent:*** induction only (6 mo): received LHRHa (Leuprorelin acetate 11.25 mg, 3-mo depot, SC or IM) plus CPA 200 mg/day orally was administered for the first 4 weeks to prevent tumor flare.
	***Continuous:*** LHRHa
De Leval 2002 [51]	***Intermittent:*** induction only (3-6 mo): flutamide (250 mg, 3 times, daily) for 15 days. This therapy was followed by flutamide and goserelin acetate (3.6 mg, monthly).
	***Continuous:*** goserelin plus flutamide (250 mg orally every 8 hours) without interruption.
Langenhuijsen 2008/2011 (*TULP Trial*) [52,53]	***Intermittent:*** induction only (6 mo): Buserelin depot 6.6 mg, a 2-monthly SC plus nilutamide 300 mg (once a day for the first 4 weeks and 150 mg daily thereafter).
	***Continuous:*** buserelin depot plus nilutamide
Miller 2007 [56]	***Intermittent:*** induction only (6 mo): goserelin plus bicalutamide
	***Continuous:*** goserelin plus bicalutamide
Mottet 2012 *(TAP 22 Trial)*[50]	***Intermittent:*** induction only (6 mo): leuprorelin SR 3.75 mg, SC every 28 days and flutamide, one 250 mg tablet, three times daily.
	***Continuous:*** leuprorelin and flutamide continued until disease progression or study end.
Verhagen 2008/2013 [48,49]	***Intermittent:*** induction only (3-6 mo): CPA 100 mg three times daily
	***Continuous:*** CPA 100 mg thrice daily.
Hering 2000 [54]	***Intermittent:*** induction only (10.5 mo): CPA 200 mg/day orally
	***Continuous:*** CPA 200 mg/day orally
Irani 2008 [57]	***Intermittent:*** induction only (6 mo): goserelin 10.8 mg 3-mo depot and flutamide 250 mg three times daily and resumed 6 mo later
	***Continuous:*** goserelin and flutamide 250 mg three times daily continued without interruption
Silva 2013 *(SEUG 9901 Trial)*[58]	***Intermittent:*** induction only (3 mo): CPA 200 mg/d for 2 weeks followed by monthly depot injections of triptoreline plus 200 mg of CPA daily and restarted monotherapy with CPA 300 mg/d in the progression
	***Continuous:*** CPA 200 mg/d for 2 weeks followed by monthly depot injections of triptoreline plus 200 mg of CPA daily.

*Abbreviations*: *Mo* months, *CPA* cyproterone acetate, *LHRHa* luteinizing hormone–releasing hormone agonist, *SC* subcutaneous, *IM* intramuscular injection.

Two studies [48,49,54] did not use luteinizing hormone–releasing hormone agonist (LHRHa) or orchiectomy. These studies used cyproterone acetate (CPA) with doses between 300-400 mg/day orally. Overall, goserelin was the most used LHRHa, followed by leuprorelin (Table 3).

Evaluation of OS was the primary endpoint in 4 studies [8-10,50,55,58] and TTP or time to androgen-independent was the primary endpoint in 7 studies [1,11,12,44-47,50-54,56,57] (Table 2). Five studies [1,8-10,44-47,55,58] were designed to evaluate the non-inferiority of IAD compared to CAD (Table 4).

**Table 4:** Efficacy analysis in the trials included in the meta-analysis

**Study**	**Arm**	**Design of study**	**Time to progression or time to castration-resistant disease**	**Cancer-specific survival**	**Overall survival (95% CI)**
Crook 2012 [9,10]*(CIC-CTG PR.7 Trial)*	CAD	Non-inferiority	10 years	HR: 1.18 (0.90-1.55)	9.1 years
	IAD	(HR <1.25)	9.8 years	p = 0.24	8.8 years
			HR: 0.80 (0.67-0.98) p = 0.024		HR: 1.02 (0.86-1.21) for non-inferiority (IAD *vs* CAD ≥1.25) = 0.009
Calais da Silva 2009/2011 [44,45]*(SEUG Trial)*	CAD	Non-inferiority	HR: 0.81 (0.63-1.05) favoring CAD	HR: 1.27 (0.98-1.64)	HR: 0.96 (0.80-1.14) favoring CAD
	IAD	(<30%)			
Hussain 2013 [8,55]*(SWOG 9346 Trial)*	CAD	Non-inferiority	NR	NR	5.8 years
	IAD	(HR <1.20)			5.1 years
					HR: 1.10 (0.97-1.25)
Salonen 2012/2013 [11,12]	CAD	Compare the efficacy	30.2 months	44.3 months	45.7 months
*(FinnProstate Trial VII)*	IAD		34.5 months	45.2 months	45.2 months
			HR: 1.08 (0.90-1.29) favoring IAD	HR: 1.17 (0.91-1.51) favoring IAD	HR: 1.15 (0.94 -1.4) favoring IAD
Tunn 2012 [1,46,47]*(EC507 Trial)*	CAD	Non-inferiority	16 risk of progression	NR	NR
	IAD		37 risk of progression		
			p = 0.853		
			HR: 0.97 (0.68-1.38) &		
De Leval 2002 [51]	CAD	Compare the efficacy	14.4 months	4 (12.1%) deaths	NR
	IAD		25.7 months	2 (5.7%) deaths	
			HR: 0.57 (0.07-4.64) &	NS	
				HR: 0.46 (0.09-2.35) &	
Langenhuijsen 2008/2011 [52,53]	CAD	Compare the efficacy	24.1 months^#^	NR	NS
(*TULP Trial*)	IAD		18 months		
			NS		
Miller 2007 [56]	CAD	Compare the efficacy	11.5 months	NR	53.8 months
	IAD		16.6 months		51.4 months
			p = 0.1758		p = 0.658
			HR: 0.69 (0.41-1.16)^&^		HR: 1.04 (0.86-1.28)^&^
Mottet 2012 [50]*(TAP 22 Trial)*	CAD	Compare the efficacy	15.1 months	NR	52 months
	IAD		20.7 months		42.2 months
			p = 0.74		p = 0.75
			HR: 0.88 (0.63-1.4)^&^		HR: 1.14 (0.74-1.77)^&^
Verhagen 2008/2013 [48,49]	CAD	Compare the efficacy	NS	NS	NS
	IAD				
Hering 2000 [54]	CAD	Compare the efficacy	20.1 months	2 (11.1%) deaths	NR
	IAD		NR	2 (8%) deaths	
			NS	NS	
				HR: 0.70 (0.09-5.44) &	
Irani 2008 [57]	CAD	Compare the efficacy	HR: 1.1 (0.6-1.8) p = 0.3 favoring IAD	HR: 0.6 (0.2–1.6) p = 0.12 favoring CAD	HR: 0.6 (0.3–1.3) p = 0.06 favoring CAD
	IAD				
Silva 2013 [58]*(SEUG 9901 Trial)*	CAD	Non-inferiority	HR: 1.16 (0.93-1.47)	HR: 1.0 (0.76-1.32)	HR: 0.90 (0.76-1.07)
	IAD	(HR < 1.20)			

*Abbreviations*: *ITT* intention-to-treat, *HR* Hazard ratios, *Mo* months, *NS* not significant, *IAD* intermittent androgen deprivation, *CAD* continuous androgen deprivation.

^#^No hazard ratio or *P* value reported.

^&^Calculated the meta-analytic survival curves as suggested by Parmar et al. [18].

More details on the treatment modality, follow-up, number of patients and primary endpoint in the 13 trials included in this analysis are summarized in Tables 2 and 3.

The efficacy analysis was summarized in Table 4.

### Meta-analysis

**Figure 2:**
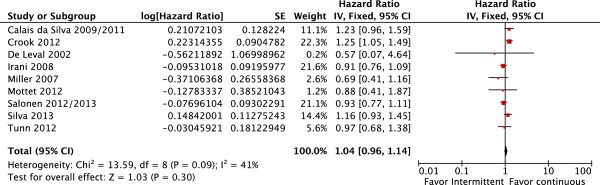
Time-to-progression: comparative effect of intermittent versus continuous androgen deprivation in prostate cancer.

Overall, TTP was similar in patients who received IAD or CAD (fixed effect: HR = 1.04; CI 95% = 0.96 to 1.14; p = 0.3), with high moderate heterogeneity (Chi^2^ = 13.59, df = 8 [P = 0.09]; I^2^ = 41%) (Figure 2).

**Figure 3:**
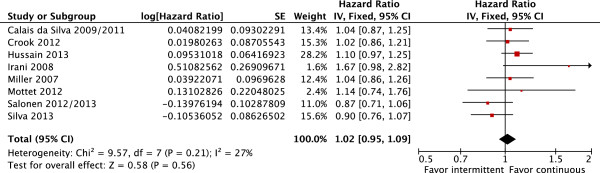
**Overall survival.** Comparative effect of intermittent versus continuous androgen deprivation in prostate cancer.

**Figure 4:**
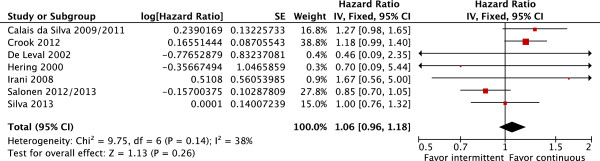
**Cancer-specific survival.** Comparative effect of intermittent versus continuous androgen deprivation in prostate cancer.

OS and CSS were also similar in patients treated with IAD or CAD (OS: fixed effect: HR = 1.02; CI 95% = 0.95 to 1.09; p = 0.56 with moderate heterogeneity Chi^2^ = 9.57, df = 7, P = 0.21, I^2^ = 27% and CSS: fixed effect: HR = 1.06; CI 95% = 0.96 to 1.18; p = 0.26 also with moderate heterogeneity Chi^2^ = 9.75, df = 6, P = 0.14, I^2^ = 38% (Figures 3 and 4, respectively).

In order to explore this heterogeneity, the study by Salonen et al. [11,12] was removed from the survival analysis, since patients treated in the IAD arm had lower levels of baseline PSA than patients on CAD group (IAD: mean PSA 116.0 ± 173.4 ng/ml; CAD: mean PSA 186.3 ± 454.4 ng/ml). Regarding OS, results remain similar between the groups (fixed effect: HR = 1.04; CI 95% = 0.96 to 1.12; p = 0.32) while there was a decrease in heterogeneity (Chi^2^ = 6.87, df = 5, P = 0.23, I^2^ = 27%). Furthermore, CSS was favorable to CAD (fixed effect: HR = 1.16; CI 95% = 1.02 to 1.31; p = 0.02; NNT = 6) with no heterogeneity (Chi^2^ = 3.51, df = 5, P = 0.62, I^2^ = 0%).

Four studies [8,48-50,54,55] included only patients with metastatic disease (or ≥90% metastatic). In this subgroup of patients, only 1 study [50] reported TTP data and another [54] reported cancer-specific survival data, so a meta-analysis was not feasible. Two studies [8,50,55] reported OS data, which permitted meta-analysis. OS was also similar in patients treated with IAD or CAD (fixed effect: HR = 1.10; CI 95% = 0.98 to 1.24; p = 0.11) with no heterogeneity (Chi^2^ = 0.02, df = 1, P = 0.88, I^2^ = 0%).

Two studies [1,9,10,46,47] included only patients with recurrent disease after definitive local therapy (prostatectomy or radiotherapy). In this subgroup, TTP was favorable to patients who received CAD (fixed effect: HR = 1.19; CI 95% = 1.01 to 1.39; p = 0.03), with moderate heterogeneity (Chi^2^ = 1.57, df = 1 [P = 0.21]; I^2^ = 36%). However, when the random-effects model analysis was performed, no significant difference was detected (random effects: HR = 1.16, CI95% = 0.92 to 1.45; p = 0.22). It was not possible to perform a meta-analysis for OS and CSS, since only Crook et al. [9,10] reported these data.

One study [58], on re-introduction of treatment in IAD group, used a hormone therapy scheme (cyproterone 300 mg/d) that was different from the one used in the CAD group (LHRHa: triptoreline 11.25 mg plus cyproterone 200 mg/d). When OS analysis was performed without this study, the results remained similar between IAD and CAD (fixed effect: HR = 1.04; CI 95% = 0.97 to 1.12; p = 0.25; with no heterogeneity: Chi^2^ = 7.08, df = 6, P = 0.31, I^2^ = 15%).

In most studies [8-10,44,48-50,55] baseline quality-of-life scores were similar in both groups for the majority of items, with no clinically significant differences. Sexual activity scores appeared to be favorable in the IAD group (Table 5).

**Table 5:** Significant differences in quality of life (QoL)

**Study**	**EORTC quality-of-life core questionnaire (QLQ-C30) and the EORTC Prostate Cancer Module**
Crook 2012 [9,10]*(CIC-CTG PR.7 Trial)*	***Intermittent (better):*** hot flashes, desire for sexual activity, and urinary symptoms
	***Continuous: -***
Calais da Silva 2009/2011 [44,45]*(SEUG Trial)*	***Intermittent (better):*** sexual function
	***Continuous (better):*** emotional domain, nausea and vomiting, severity of insomnia
Hussain 2013 [8,55]*(SWOG 9346 Trial)*	***Intermittent (better):*** erectile function and mental health at 3 months but not thereafter
	***Continuous:*** -
Salonen 2012/2013 [11,12]*(FinnProstate Trial VII)*	***Intermittent (better):*** sexual function
	***Continuous:*** -
Tunn 2012 [1,46,47]*(EC507 Trial)*	***Intermittent:*** NR
	***Continuous:*** NR
De Leval 2002 [51]	***Intermittent:*** NR
	***Continuous:*** NR
Langenhuijsen 2008/2013 [52,53] (*TULP Trial*)	***Intermittent:*** NS
	***Continuous:*** NS
Miller 2007 [56]	***Intermittent (better):*** sexual activity appeared to be favorable in the intermittent
	***Continuous:*** -
Mottet 2012 [50]*(TAP 22 Trial)*	***Intermittent:*** NS
	***Continuous:*** NS
Verhagen 2008/2013 [48,49]	***Intermittent (better):*** symptom and potency scales
	***Continuous:*** -
Hering 2000 [54]	***Intermittent (better):*** erectile function
	***Continuous:*** -
Irani 2008 [57]	***Intermittent (better):*** ability to have and maintain an erection.
	***Continuous:*** -
Silva 2013 [58]*(SEUG 9901 Trial)*	***Intermittent (better):*** sexual activity
	***Continuous:*** -

*Abbreviations*: *EORTC* European Organization for Research and Treatment of Cancer, *NS* not significant, *NR* not reported.

**Figure 5:**
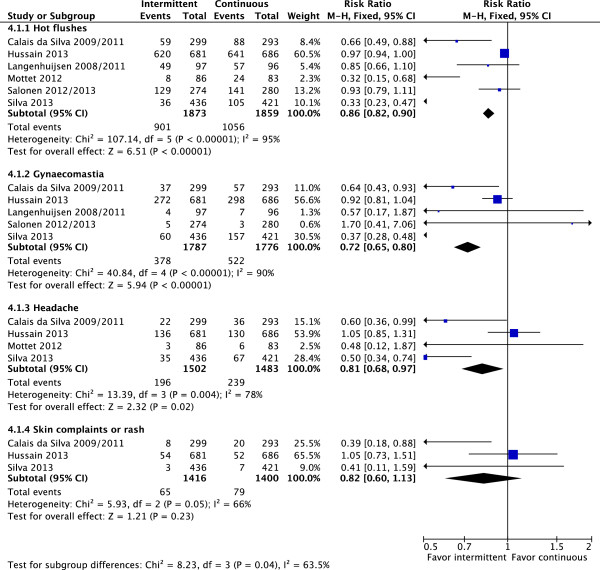
**Toxicities.** Comparative effect of intermittent versus continuous androgen deprivation in prostate cancer.

The prevalence of adverse events of androgen deprivation such as hot flushes (fixed effect: RR = 0.86; CI 95% = 0.82 to 0.90; p < 0.00001), gynecomastia (fixed effect: RR = 0.72; CI 95% = 0.65 to 0.80; p < 0.00001) and headache (fixed effect: RR = 0.81; CI 95% = 0.68 to 0.97; p-0.02) were more frequent in patients treated with CAD, with high heterogeneity levels (Figure 5). As planned, a random-effects model analysis was performed to better explore this heterogeneity. In this analysis, with exception of hot flushes (RR = 0.65; CI 95% = 0.45 to 0.95; p = 0.03; NNH = 10), adverse events were similar in both groups (gynecomastia: RR = 0.66; CI 95% = 0.39 to 1.13; p = 0.13 and headache: RR = 0.68; CI 95% = 0.42 to 1.10; p = 0.11).

Mortality secondary to cardiovascular events was report in only 4 studies [9-12,44,45,58]. Results of this analysis were favorable to the group treated with IAD (fixed effect: RR = 0.80; CI 95% = 0.67 to 0.94; p = 0.007; NNH = 33) however with high heterogeneity levels (Chi^2^ = 6.67, df = 3 [P = 0.08]; I^2^ = 55%).

According to funnel plot analysis [23] the possibility of publication bias was low for all of the outcomes. When funnel plot shows asymmetry, there is a possibility of bias and despite its limmitations, this method is widely used by authors.

## Discussion

Currently, many oncology guidelines do not recommend the use of IAD for patients with metastatic hormone-sensitive prostate cancer [36,59]. But it is important to note that these guidelines date back to 2007. Guidelines from the European Association of Urology (EUA) state that IAD is now widely offered to patients with prostate cancer and should no longer be regarded as investigational [60,61].

Two other meta-analysis published earlier [62,63] showed that both treatments had similar results regarding OS and CSS. Analysis of OS was performed with 4 studies [8-12,44,45,55] in the Niraula et al. meta-analysis [62] and 7 studies [8-12,44,45,50,52,53,55,57] in Tsai et al. [63]. CSS analysis was performed with 3 [9-12,44,45] and 6 studies [8-12,44,45,51,55,57] respectively.

In this present meta-analysis, we included a larger number of studies. When HRs were not directly reported in the original study, they were estimated indirectly by using the reported number of events and the corresponding *P* value for the log-rank statistics, or by reading survival curves as suggested by Parmar et al. [18]. To reduce reading errors, original survival curves were digitalized and enlarged, and data extraction was based on reading electronic coordinates for each point of interest, as described by DeLaurentiis in another meta-analysis [64].

In the final OS and CSS analyses we included 8 and 7 studies respectively. The induction time of androgen deprivation was, in general, 3-6 months. Two studies were available only in abstract form [48,49,56]. Overall, goserelin was the most used LHRHa, followed by leuprorelin. Only two studies [48,49,53] did not used luteinizing hormone–releasing hormone agonist (LHRHa) or orchiectomy. These studies used cyproterone acetate (CPA).

In this meta-analysis, randomized studies included heterogeneous groups of patients (locally advanced, metastatic or recurrent). It was not possible to perform the proper analysis of subgroups of patients (i.e. by Gleason score, initial PSA levels, minimal or extensive metastatic disease) because results for such sets were not published in the studies. As to overall survival, our results reinforce the equivalent efficacy of CAD and IAD, regardless of previous treatments.

Apropos of CSS, despite the overall analysis demonstrating only a trend towards treatment with CAD, the results must be interpreted with caution. As mentioned before, in one of the studies [11,12] the baseline PSA levels were lower on the IAD arm, suggesting that these patients had less extensive disease. Once this trial was excluded from the analysis, results favored the group treated with CAD; therefore we cannot exclude the possibility that IAD may carry a higher risk of death by cancer.

Recently, a large prospective Danish study, including over 30,000 patients, investigated the relationship between androgen deprivation therapy (ADT) and cardiovascular diseases such as myocardial infarction (MI) and stroke in men with prostate cancer [65]. The authors found that patients treated with medical endocrine therapy had an increased risk for MI and stroke with adjusted HRs of 1.31 (95% confidence interval [CI], 1.16-1.49) and 1.19 (95% CI, 1.06-1.35), respectively, compared with nonusers of ADT. However, androgen deprivation secondary to orchiectomy did no increase the risks for MI (HR: 0.90; 95% CI, 0.83-1.29) or stroke (HR: 1.11; 95% CI, 0.90-1.36). Nevertheless, the conclusions might have been affected, by the lack of information on prognostic lifestyles.

In this meta-analysis, mortality secondary to cardiovascular diseases, despite the heterogeneity found and the paucity of studies reporting this outcome, was favorable to the group treated with IAD. This aspect might have influenced the similar results achieved in OS.

In the event of biochemical recurrence after prostatectomy, when androgen deprivation is indicated, the best candidates for IAD are patients who reach PSA values <0.5 ng/mL after the induction time, according to a randomized study that was considered pure (i.e which included only patients with recurrence after prostatectomy) [1,46,47]. For biochemical recurrences after radiotherapy, the best candidates for IAD are patients who reach PSA levels <4 ng/mL after the induction time [9,10]. Only in these situations (recurrence after radiotherapy or prostatectomy), TTP seems to be favorable to patients receiving CAD. In locally advanced and/or metastatic tumors, the best candidates for IAD are patients who reach PSA values <4 ng/mL after induction time.

Although TTP analysis was performed in this meta-analysis, the results should be evaluated with caution since criteria for progression were different among the included studies.

Patients with a lower burden of disease (i.e. less extensive diseases) and without co-morbidities may be the ones achieving most benefits with IAD treatment.

The implementation of guidelines to assess, monitor and reduce known risk factors for cardiovascular disease may influence the results of overall survival in patients treated with CAD.

Regarding quality of life, differences were not clinically significant in most studies. But it was observed that sexual activity scores seemed to be higher in patients treated with IAD, and the incidence of hot flushes was lower.

Furthermore, it is important to note that IAD modality offers an economic benefit with reduction of pharmaceutical costs during “off-therapy” [62]. Economic analysis is not the aim of this investigation. The studies included failed to report the costs of both modalities (intermittent and continuous). Niraula et al. [62], analyzing only drug costs, estimated that the median cost savings with IAD would be 48%. Hering et al. [54], also based only on the average drug costs, demonstrated that the cost of treatment in IAD arm was approximately 50% lower that in the CAD, over 48 months.

## Conclusions

Overall survival was similar between IAD and CAD in patients with locally advanced, recurrent or metastatic hormone-sensitive prostate cancer. Data on CSS are weak and the benefits of IAD on this outcome remain uncertain. Impact in QoL was similar for both groups, however, sexual activity scores were higher and the incidence of hot flushes was lower in patients treated with IAD.

## Competing interests

The authors declare that they have no competing interests.

## Authors’ contributions

Systematic review and meta-analysis TEAB, OC. Identification of studies, critical evaluation and discussion. RBDR, ACLP, UF, MVS, FFHBT, TEAB. All authors read and approved the final manuscript.

## Pre-publication history

The pre-publication history for this paper can be accessed here:

http://www.biomedcentral.com/1471-2490/14/9/prepub
